# Sanatoria Treatment and the Poor Law

**Published:** 1914-02-14

**Authors:** 


### The Hospital

The Workers' Newspaper of Administrative Medicine and Institutional Life, Administration, National Insurance and Health.

No. 1442, Vol. LV. SATURDAY, FEBRUARY 14, 1914. PRICE ONE PENNY

### SANATORIA TREATMENT AND THE POOR LAW.

Although large sums of money have been spent in building and maintaining special hospitals and sanatoria, many of the tuberculous poor remain, for whom the Poor Law has to provide. The Poor-Law system has been adapted to meet the needs of this work in a manner perhaps not duly recognised. Not only have wards been provided and set aside for cases of advanced disease (large numbers of which have finally drifted into our Poor-Law hospitals and infirmaries), but the more progressive Boards of Guardians have found sanatorium accommodation for suitable patients, have afforded relief to their dependants, and have taken the question of prevention of the spread of the disease into consideration. The failure of the work done has been due to the utter absence of after-care and of supervision as regards maintenance and housing. Many years ago a conference of Poor-Law authorities was convened and met in London to discuss the question of providing sanatorium accommodation, and the position in the matter of the Metropolitan Asylums Board. The conference led to no definite action; principally because it was recognised that any provision made would be of such a character that very large numbers of patients who had never sought Poor-Law relief and treatment would readily avail themselves of it. An enormous expenditure would undoubtedly have fallen upon the ratepayers had sanatorium treatment been then provided even for consumptive patients in London by the Boards of Guardians. It is to be remembered that under the Poor Laws provision is made primarily for dealing with the destitute and the infirm; the extension of the scope of the work to include widespread hospital and other medical treatment was not contemplated until recent years, and has in great measure resulted from the absence of any other authority possessed of the necessary powers and capable of raising money. The health authorities, although possessed of certain powers under the Public Health Acts, have been able in the past to take but little part in arranging for the treatment of tuberculosis.

Upon the passing into law of the National Insurance Act, 1911, there necessarily followed an organisation, entirely new and State aided, for the treatment of tuberculosis. It is unnecessary for us to recapitulate in The Hospital the steps which led to the present position of this organisation, and which have been fully detailed in previous issues. Suffice it to say that arrangements for the treatment of insured patients *must* be made by the Insurance Committees and that similar arrangements in the case of consumptive dependants of the insured *may* be made by them; further, that the scheme for treatment (generally carried out by the Health Department of the County Councils) is being extended to all patients, whether adults or children, suffering from tuberculosis in any form. There is one important exception with regard to the "arrangements" to be made by the Insurance Committees—namely, that such arrangements are not to be made with Poor-Law authorities.

The persons for whom treatment is provided are, therefore: (*a*) insured persons, male or female, and (*b*) uninsured persons, consisting of dependants of the insured and those not so dependent—adults or children. The costs incurred in treating class (*a*) will be defrayed from the funds of the Insurance Committees. In cases where the Committee does not undertake the treatment of dependants the costs incurred in treating class (*b*) will be entirely defrayed by the County Councils.

It is to be observed that the scheme is for the provision of *treatment*. Such treatment ("sanatorium benefit") may be divided into: (*a*) sanatorium treatment; (*b*) hospital treatment, for observation or in advanced cases; (*c*) dispensary treatment; and (*d*) domiciliary treatment. Very considerable progress has been made in the development of means for providing sanatorium and hospital treatment, many institutions have been approved, and much building is in progress, or is contemplated; the accommodation so provided will be available alike for the insured and for the uninsured, as will be the advice and treatment afforded by the dispensaries. It is obvious that, whilst treatment in a sanatorium or hospital may be provided in the case of uninsured patients suited for such treatment, "domiciliary" treatment for them, when unfit to attend at a dispensary or in emergencies, is their own concern.

Very serious is the question of the maintenance of tuberculous patients and of their dependants. Stress has been laid upon the fact that the provision made has in view the *treatment* of tuberculosis. How intimately related are the questions of such treatment and that of maintenance is shown by the importance which Poor-Law provision has assumed in the treatment of tuberculosis. It is true that an insured patient may receive "sick benefit," that such benefit may be applied in assisting dependants whilst the patient is under institutional treatment, and that a limited allowance for extra nourishment may be made to the patient whilst receiving domiciliary or dispensary treatment. Nevertheless, the district Insurance Committees and their After-Care Sub-Committees have no funds for distribution and the relief of destitution, or, in other words, the granting of "out relief," whether for the benefit of a patient or of the dependants, or both, remains a Poor-Law question even in the case of the tuberculous; and from the Poor-Law authorities the Insurance Committees are, in some degree, specifically dissociated. Some Poor-Law Boards have, apparently, imagined that treatment in, and responsibility in cases of, tuberculous disease are to be taken entirely out of their hands; this supposition is unfounded, as maintenance (which is the concern of the Poor Law) must sometimes be allied to treatment. The powers for treatment now under consideration are largely permissive.

It is impossible to avoid the conclusions that, under existing conditions and for some time to come at least, funds for maintenance must be found for tuberculous patients and their dependants, and that no scheme for treatment can be entirely dissociated either from charitable agencies or from Poor-Law relief.

